# Comparative study on mechanical and antibacterial properties of nanoparticle-reinforced orthodontic composites

**DOI:** 10.6026/973206300220251

**Published:** 2026-01-31

**Authors:** Vidyesh Nadkerny, Thimmaiah UK, Manorama Namdev Wakle, Waleed Ali F Alshadidi, Ravi Jhamb, Anshuman Mishra, Santosh Kumar

**Affiliations:** 1 Department of Orthodontics and Dentofacial Orthopaedics, Daswani Dental College & Research Centre, Kota, Rajasthan; 2 Department of Orthodontics, Farooqia Dental College & Hospital, Umar Khayam Road, Eidgah, Tilak Nagar, Mysuru, Karnataka, India; 3 Department of Orthodontics and Dentofacial Orthopaedics, Yogita Dental college & Hospital, Khed, Maharashtra, India; 4 Department of Endodontics, Mazayalian Dental complex clinic, Abha, Saudi Arabia; 5 Department of Orthodontics and Dentofacial Orthopaedics, Pacific Dental College and Hospital, Debari, Udaipur, Rajasthan; 6 Department of Orthodontics and Dentofacial Orthopaedics, Hi-Tech Dental College and Hospital, Bhubaneswar, India; 7 Department of Periodontology & Implantology, Karnavati University, Uvarsad, Gandhinagar, Ahmedabad, Gujarat, India

**Keywords:** Orthodontic composite, nanoparticle, zirconia, silver, shear bond strength, antibacterial, white spot lesions

## Abstract

Bond failure and white spot lesion are continuing issues during fixed orthodontic therapy. Therefore, it is of interest to compare mechanical and antibacterial properties of four orthodontic composite such as zirconia-reinforced, silver-reinforced and hybrid nanoparticle composites. The ZrO_2_ and the hybrid groups had high shear bond and flexural strengths when compared to the commercial control and the AgNP-containing groups were very effective in the anti-bacterial action against *S. mutans*. The composite hybrid was a unique compound that was to offer a high mechanical performance and the strong antibacterial action. Altogether, zirconia-silver hybrid material can be discussed as an effective multifunctional substance that will help avoid debonding and enamel demineralization processes during orthodontic treatment.

## Background:

Fixed appliances used to treat orthodontics depend on the ability of brackets to bond to enamel in teeth with resin-based composites. The clinical effectiveness of this procedure in the long-term is conditional on the adhesive capacity to resist the masticatory and orthodontic forces that are complicated by nature and occur in the oral cavity [1]. There are adhesive failure and bracket debonding which are typical clinical issues that require extra appointments, raise costs and time of treatment. Thus, increasing the mechanical characteristics, especially shear bond strength (SBS) of orthodontic composites is one of the main goals of research in dental materials. In addition to mechanical integrity, there is a second, equally important issue, which is the threat of a higher risk of enamel demineralization, which is clinically presented as white spot lesions (WSLs). The complex structure of orthodontic brackets, archwires and ligatures form many areas of stagnation which are preferred by accumulation of dental plaque [2]. Such pathologic biofilms which are predominantly comprised of acidogenic bacteria such as *Streptococcus mutans* generate organic acids which demineralize the adjacent enamel surface causing unsightly and usually irreversible WSLs in up to 50% of patients [3]. Creation of orthodontic adhesives with inbuilt antibacterial properties can be significant in eliminating this widespread iatrogenic outcome. Over the last few years, nanotechnology has become a revolutionary technology in dental materials science, providing a route to the creation of multi-functional or multi-purpose biomaterials that are considered as intelligent or smart [4]. In the case of a polymer, the incorporation of nano-sized particles into the polymer matrix can be used to customize the material at the molecular scale. As an example, nano-fillers like zirconia (ZrO_2_) or silica (SiO_2_) have been demonstrated to enhance the mechanical performance of dental composites by increasing the spread of loads and by preventing crack propagation [5, 6]. These materials are very strong and biocompatible and can therefore be considered as good candidates to mechanical reinforcement. At the same time, research has been more than thorough on development of antibacterial dental materials by integrating agents that are able to suppress or eliminate the formation of biofilms. AgNPs are especially interesting because their antimicrobial activity is powerful and broad-spectral with the lowest concentrations [7]. The mechanisms suggested entail the discharge of silver ions, which break cell membranes in bacteria, denature important enzymes and distort DNA replication [8]. This has also been done with other agents e.g. zinc oxide (ZnO) nanoparticles, quaternary ammonium methacrylate (QAM) compounds amongst others [9]. There is a possibility that the introduction of one kind of nanoparticle would undermine the advantage provided by the other. Therefore, it is of interest to report comparative evaluation of the mechanical (shear bond strength and flexural strength) and antibacterial properties of a new orthodontic composite with zirconia nanoparticles, silver nanoparticles and hybrid combination of these nanoparticles versus a conventional commercial composite becomes of interest.

## Materials and Methods:

## Study design:

This was an *in vitro* experimental study designed to compare the properties of four orthodontic composite groups.

## Preparation of experimental composites:

An experimental light-curable resin matrix was prepared by mixing 70 wt% Bisphenol A-glycidyl methacrylate (Bis-GMA) and 30 wt% triethylene glycol dimethacrylate (TEGDMA). Camphorquinone (0.5 wt%) and dimethylaminoethyl methacrylate (0.5 wt%) were added as the photo-initiator system. This unfilled resin matrix served as the base for the experimental groups.

## Four composite groups were prepared:

[1] Group 1 (Control): A commercially available orthodontic composite (Transbond XT, 3M Unitek, USA) was used as the control.

[2] Group 2 (ZrO_2_): The experimental resin matrix was filled with 40 wt% silane-treated zirconia nanoparticles (ZrO_2_, mean particle size 50 nm, Sigma-Aldrich).

[3] Group 3 (AgNP): The experimental matrix was filled with 1 wt% silver nanoparticles (AgNP, mean particle size 25 nm, Sigma-Aldrich) and 39 wt% silane-treated inert glass fillers (mean particle size 1 µm) to maintain a constant total filler load of 40 wt%.

[4] Group 4 (Hybrid): The experimental matrix was filled with 39 wt% silane-treated zirconia nanoparticles (50 nm) and 1 wt% silver nanoparticles (25 nm).

Nanoparticles were dispersed in the resin matrix using a high-speed mechanical mixer followed by ultrasonication to ensure homogenous distribution.

## Mechanical property testing:

[1] Shear Bond Strength (SBS) test: Eighty sound human premolars, extracted for orthodontic purposes and free of caries or cracks, were collected under an approved ethics protocol. The buccal surfaces were cleaned and polished. The teeth were randomly divided into the four groups (n=20/group). Stainless steel orthodontic brackets (MBT 0.022-slot) were bonded to the acid-etched (37% phosphoric acid) and primed buccal surfaces according to the manufacturer's instructions for the control group and using a standardized technique for the experimental groups. All samples were light-cured for 40 seconds. After bonding, the samples were stored in deionized water at 37°C for 24 hours. SBS was tested using a universal testing machine (Instron) with a knife-edge blade at a crosshead speed of 1.0 mm/min until failure. The force at failure (N) was recorded and converted to megapascals (MPa) by dividing by the bracket base area.

[2] Flexural Strength (FS) Test: For each group, 15 bar-shaped specimens (25 mm x 2 mm x 2 mm) were prepared using a stainless-steel mold according to ISO 4049 standards. The composite was placed in the mold, covered with a Mylar strip and light-cured from both sides. After removal from the mold, specimens were stored in deionized water at 37°C for 24 hours. A three-point bending test was performed using the universal testing machine with a 20 mm span and a crosshead speed of 0.5 mm/min. FS was calculated in MPa.

## Antibacterial activity assessment:

[1] Bacterial strain and culture: *Streptococcus mutans* (ATCC 25175) was used. The strain was cultured in Brain Heart Infusion (BHI) roth at 37°C in a 5% CO_2_ atmosphere.

[2] Biofilm formation assay: For each of the four groups, 15 sterile disc-shaped composite specimens (10 mm diameter, 2 mm thick) were prepared. The discs were placed in a 24-well plate. A 1 mL aliquot of *S. mutans* suspension (1 x 10^8^ CFU/mL in BHI broth with 1% sucrose) was added to each well. The plates were incubated at 37°C for 24 hours to allow biofilm formation.

[3] CFU counting: After incubation, the discs were gently rinsed with phosphate-buffered saline (PBS) to remove planktonic bacteria. Each disc was then transferred to a tube containing 1 mL of PBS and sonicated for 30 seconds to dislodge the adherent biofilm. The resulting bacterial suspension was serially diluted, plated onto BHI agar plates and incubated for 48 hours. The number of colony-forming units (CFUs) was counted and the results were expressed as log_10_ CFU/mL.

## Statistical analysis:

Data were analyzed using SPSS Statistics 26.0 (IBM Corp.). The Shapiro-Wilk test was used to check for normal distribution. A one-way analysis of variance (ANOVA) followed by Tukey's honestly significant difference (HSD) post-hoc test was used to compare the means of the four groups for SBS, FS and log CFU counts. The level of statistical significance was set at p < 0.05.

## Results:

The mean values and standard deviations for shear bond strength and flexural strength are presented in Table 1 and Table 2, respectively. The highest SBS was recorded for the ZrO_2_ group (20.5 ± 2.4 MPa), followed closely by the Hybrid group (19.8 ± 2.1 MPa). One-way ANOVA revealed a statistically significant difference among the groups (p<0.001). Tukey's post-hoc test showed that both the ZrO_2_ and Hybrid groups had significantly higher SBS than the Control group (16.2 ± 1.9 MPa) and the AgNP group (15.5 ± 2.3 MPa) (p<0.01). There was no significant difference between the Control and AgNP groups (p=0.52). For flexural strength, the ZrO_2_ group exhibited the highest value (112.5 ± 9.8 MPa), followed by the Hybrid group (108.4 ± 11.2 MPa). The AgNP group showed the lowest FS (81.7 ± 9.1 MPa). The differences among all groups were statistically significant (p<0.001). The ZrO_2_ and Hybrid groups were significantly stronger than the Control (85.3 ± 8.5 MPa) and AgNP groups. The results of the antibacterial assay are shown in Table 3. The Control and ZrO_2_ groups supported substantial bacterial growth, with no significant difference between them (p=0.91). In contrast, both the AgNP and Hybrid groups demonstrated powerful antibacterial effects, with significantly lower bacterial counts compared to the Control and ZrO_2_ groups (p<0.001). This translated to a >2.5-log reduction in viable *S. mutans* counts. There was no statistically significant difference in the antibacterial efficacy between the AgNP and Hybrid groups (p=0.78).

## Discussion:

The results of this *in vitro* experiment prove that the implementation of a hybrid nanoparticle system can be effective to develop a multi-functional orthodontic composite with high mechanical strength as well as effective antibacterial activity. The new hybrid composite that comprised of both zirconia and silver nanoparticles performed better than the traditional composite and the single- nanoparticle experimental groups in general performance. It can be affirmed that the reinforcement effect of the zirconia nanoparticles contributed to the high increase in both shear bond strength and flexural strength of the ZrO_2_ and Hybrid groups. ZrO_2_ is nanoparticles with high surface area and nanometric size and can therefore effectively penetrate the polymer matrix as centers of stress and prevent the formation and propagation of microcracks in the polymer under load [10, 11]. Silanization of these nanoparticles is important as this will form a strong covalent bond between inorganic filler and the organic resin matrix such that efficient transfer of stress and no debonding of the filler occurs [12]. The high flexural strength in the hybrid silica-zirconia filled group indicates that 1 wt% AgNPs do not disrupt the reinforcing action of the γ-MPS-treated fillers or the polymer matrix integrity. In contrast, the AgNP group with increased zirconia content exhibited slightly reduced flexural strength and modulus compared to the control, suggesting that higher filler loading induces stress concentrations from uneven dispersion [13]. The high antibacterial effect of AgNP and Hybrid groups is of intellectual consistency with the long-reported antimicrobial effects of silver nanoparticles [7, 14]. The >2.5-log *S. mutans* viability reduction is a clinical significant kill rate of over 99.5 percent that can potentially have a significant impact on the cariogenic burden around orthodontic brackets. This is mainly facilitated by the liberation of the silver ions (Ag+), which are very reactive and cytotoxic to the bacteria amongst the nanoparticles [8]. As anticipated, ZrO_2_ group exhibited no anti-bacterial activity proving that zirconia is biologically inert in such a situation. The strong antibacterial activity of the Hybrid group validates the fact that the silver nanoparticles still works even in case of co-dispersing with a high level of zirconia nanoparticles. Clinically, the Hybrid composite is the most likely to succeed. It was able to deal with the two major clinical issues identified in the introduction. It also has high shear bond strength (19.8 Mpa) that is well beyond the acceptable clinical range of 6-8 Mpa implying a less likely occurrence of bracket debonding [15]. At the same time, the fact that it can greatly suppress *S. mutans*, the main etiological agent of caries and WSLs, has an inherent protective response to enamel demineralization. This is not only an important development compared to the traditional orthodontic adhesives which are passive [16]. There are a number of limitations that this study has. Being an *in vitro* study, it is not able to recreate the oral environment fully in its complexity and dynamics including saliva, multi- species biofilm, temperature changes and fluctuating pH. The rate of leaching silver ions, the probable discoloration of the nanoparticle- reinforced composites and the mechanical degradation during the long-term, are the areas that need investigations [17]. Furthermore, although the biocompatibility of AgNPs at low concentrations has been excellent in most studies, long-term cytotoxicity studies are needed in full before any clinical translation can occur [18]. Future studies are needed to enhance the concentrations of nanoparticles; other antibacterial agents such as QAMs and finally, *in situ* and *in vivo* clinical trials are required to confirm the potential of such interesting laboratory results.

## Conclusion:

It was found that a hybrid system of zirconia and silver nanoparticles incorporated into an experimental orthodontic composite resin produces a multi-functional biomaterial with a high level of functionality. The hybrid composite showed much improved shear bond and flexural strength over a conventional composite and, at the same time, they had strong antibacterial activity against *S. mutans*.

## Figures and Tables

**Table 1:** Shear Bond Strength (SBS) results for the four composite groups (n=20)

**Group**	**Mean SBS (MPa) ± SD**	**p-value**
Control	16.2 ± 1.9 ^b^	<0.001*
ZrO_2_	20.5 ± 2.4 ^a^	
AgNP	15.5 ± 2.3 ^b^	
Hybrid (ZrO_2_ + AgNP)	19.8 ± 2.1 ^a^	
*Superscript letters indicate statistically significant differences (p<0.05). Groups sharing the same letter are not significantly different		

**Table 2:** Flexural Strength (FS) results for the four composite groups (n=15)

**Group**	**Mean FS (MPa) ± SD**	**p-value**
Control	85.3 ± 8.5 ^c^	<0.001*
ZrO_2_	112.5 ± 9.8 ^a^	
AgNP	81.7 ± 9.1 ^c^	
Hybrid (ZrO_2_ + AgNP)	108.4 ± 11.2 ^b^	
*Superscript letters indicate statistically significant differences (p<0.05). Groups sharing the same letter are not significantly different.		

**Table 3:** Antibacterial Activity against *S. mutans* (n=15)

Group	Mean Log_10_ CFU/mL ± SD	p-value
Control	7.6 ± 0.3 ^a^	<0.001*
ZrO_2_	7.5 ± 0.4 ^a^	
AgNP	4.9 ± 0.5 ^b^	
Hybrid (ZrO_2_ + AgNP)	4.8 ± 0.6 ^b^	
*Superscript letters indicate statistically significant differences (p<0.05). Groups sharing the same letter are not significantly different		
